# TrackCC: A Practical Wireless Indoor Localization System Based on Less-Expensive Chips

**DOI:** 10.3390/s17061391

**Published:** 2017-06-15

**Authors:** Xiaolong Li, Yan Zheng, Jun Cai, Yunfei Yi

**Affiliations:** 1 The School of Computer Science and Information Security, Guangxi Key Laboratory of Trusted Software, Guilin University of Electronic Technology, Guilin 541004, China; zhengyanbeil@gmail.com; 2 Mobile E-Business Collaborative Innovation Center of Hunan Province, Key Laboratory of Hunan Province for Mobile Business Intelligence, Hunan University of Commerce, Changsha 410205, China; 3 The Department of Electrical and Computer Engineering, University of Manitoba, Winnipeg, MB R3T 5V6, Canada; jun.cai@umanitoba.ca; 4 The Department of Computer and Information Engineering, Hechi University, Hechi 546300, China; gxyiyf@hcnu.edu.cn

**Keywords:** wireless indoor localization system, signal fluctuation, discrete power outputs, pattern matching, cheap communication chip

## Abstract

This paper aims at proposing a new wireless indoor localization system (ILS), called TrackCC, based on a commercial type of low-power system-on-chip (SoC), nRF24LE1. This type of chip has only *l* output power levels and acute fluctuation for a received minimum power level in operation, which give rise to many practical challenges for designing localization algorithms. In order to address these challenges, we exploit the Markov theory to construct a (l+1)×(l+1) -sized state transition matrix to remove the fluctuation, and then propose a priority-based pattern matching algorithm to search for the most similar match in the signal map to estimate the real position of unknown nodes. The experimental results show that, compared to two existing wireless ILSs, LANDMARC and SAIL, which have meter level positioning accuracy, the proposed TrackCC can achieve the decimeter level accuracy on average in both line-of-sight (LOS) and non-line-of-sight (NLOS) senarios.

## 1. Introduction

Localization plays a key role in various business scenarios. The most famous localization system is the Global Position System (GPS). It has been widely used for outdoor localization but performs poorly in indoor environments due to blocked satellite signals. During the last ten years, indoor localization has been an important technical support in many wireless distribution systems due to: (1) its ability to position objects in places where outdoor localization systems cannot cover; and (2) too large graininess of outdoor systems to serve indoor environments with high precision requirements. Recently, indoor localization systems (ILSs) based on wireless signals have attracted researchers’ interests in a wide range of real applications, such as emergency personnel localization in a disaster area, patient tracking in a hospital and location detection of assets in a warehouse. Compared to other ILSs, e.g., vision-based localization system, wireless ILSs can simultaneously achieve low cost and high accuracy. According to the wireless infrastructure, wireless ILSs are usually classified into the following categories: Radio Frequency IDentification (RFID) ILS, WiFi ILS, Bluetooth ILS, etc. RFID ILS is capable of working for a relatively long time due to low power consumption, but the main drawback that commercial off-the-shelf readers have is that only a small reading distance of around 10 m [1] seriously limits its application fields. Bluetooth ILS [2] suffers from similar limitations. WiFi ILS can achieve a centimeter level localization precision [3,4]. However, it is required to equip high-power and relatively expensive WiFi chips with continuous AC power supplies into mobile devices and deploy a number of wireless access points. In this paper, different from conventional wireless ILSs, we will explore the localization possibility of a wireless ILS built on a low-power low-cost wireless chip, nRF24LE1, which only provides several discrete power outputs, and is not capable of measuring received signal strengths.

Wireless ILS works mainly based on four metrics. They are time of arrival (ToA), time difference of arrival (TDoA), angle of arrival (AoA) and received signal strength indicator (RSSI). Wang et al. [5] proposed a ToA-based ILS. It used DBSCAN algorithm to divide potential positions into two parts, inliers and outliers. The algorithm deleted outliers through iterative clustering, voting and filtering. Consequently, the position of the inliers gradually converged to a calculated position. Although ToA-based solutions have high accuracy in LOS scenarios, they require expensive time synchronization devices to calibrate time between transmitters and receivers. Since the time of arrival can be disrupted under NLOS scenarios, which significantly degrades localization accuracy, Pak et al. [6] proposed a ToA-based hybrid particle finite impulse response filter algorithm to mitigate NLOS effects by identifying NLOS links. A TDoA based localization approach adopts multiple receivers to record arrival times of a same source signal and uses the time difference among multiple measuring receivers for localization. Xiong et al. [7] proposed a TDoA-based ILS, called ToneTrack, to address the challenging situation, where direct path signals were blocked. Based on ToA readings from pairs of access points (APs), techniques such as triangle inequality, clustering and rejecting outlier, were adopted to yield the location estimations of mobiles. However, similar to ToA, TDoA suffers from multipath propagation in an NLOS environment, complex time asynchronization, and expensive hardware cost. Xiong et al. [8] proposed an AoA-based ILS, called ArrayTrack, which used multiple APs to form antenna arrays. It computed AoA of direct path to achieve a high accuracy. However, the difficult large-scale antenna array deployment and the rare direct path make it hard to be used in complex indoor environments.

RSSI methods use signal strength to estimate distances between transmitters and receivers. It is based on the signal-fading model that, as the distance increases, the RSSI of radio signal decreases monotonically. Since changes in the RSSI value have a direct impact on the estimation of distance, the positioning accuracy has high sensitivity to the link quality. Thus, there are many existing algorithms in order to identify, smooth and even exclude impacts from poor link quality. Huang et al. [9] used the Kalman Filter and a traditional geometric method for location estimation. The tags used could continuously get accurate RSSI values in the range from 0 to 255. To achieve more precise localization, the basic linear Kalman filter smoothed the fluctuations of RSSI rather than removing them completely. However, different from conventional RSSI-based ILSs with an assumption that equipped chips can measure received signal strength, some practical chips, especially those with low prices, only output discrete power levels. As referred to LANDMARC in [10], the reader only supplied eight power levels. Since the physical distance cannot be computed by using power level directly, LANDMARC developed a fingerprint-based approach to identify a reference point with a uniquely marked vector of power levels that the readers detected from a reference tag on the same point. When a target tag goes into the communication range of readers, the system receives a power level vector between the target and these readers, and uses this vector by matching to calculate the current position of the target. Another ILS, called SAIL [11], utilized the feature that nearby reference nodes have similar signal strengths. By adopting the *k*-means clustering algorithm, the corresponding cluster of an unknown node was chosen. According to the similarity of signal strengths, the weight values of reference nodes within the cluster were further assigned. Finally, the position of the node was calculated using a linear-least-square algorithm. Different from both algorithms above, in this paper, we focus on addressing the fluctuation issue of power levels caused by inexpensive communication chips nRF24LE1 and developing a similarity match scheme of the received minimum power level vectors to improve the localization accuracy.

This paper proposes a new wireless indoor localization system, called TrackCC, which is built on a commercial type of low-power low-cost chip, nRF24LE1. This chip supports four distinct programmable power levels. They are −18 dBm , −12 dBm , −6 dBm, and 0 dBm, indicated as PL-1, PL-2, PL-3, and PL-4, respectively. Since the chip is not able to measure the RSSI value of the radio signal, traditional RSSI-based ILS solutions are not feasible in this case. In addition, during the practical operation of TrackCC, we notice that there are serious nonlinear fluctuations in received minimum power levels, which may be caused by environmental noise interference, unstable cheap hardware, dynamic movements of obstruction, etc. All of them impose big challenges in achieving high localization accuracy. Since the received minimum power level may not be necessarily proportional to the distance in NLOS situations, we construct a signal map with grid center points as the fingerprints of the whole area. Each fingerprint consists of a vector of received minimum power levels. The proposed TrackCC consists of two phases. The offline phase is used to remove the fluctuations in power levels and form the radio map, and the online phase exploits the proposed priority-based pattern matching algorithm to find the nearest grid center point by selecting the most similar match on power level vectors between fingerprints and that sent by the unknown node. We conduct extensive experiments to demonstrate the effectiveness and superiority of the proposed TrackCC. The major contributions of our work are summarized as follows:

(1) Based on a commercial low-cost low-power system-on-chip, nRF24LE1, the proposed system can achieve decimeter-level accuracy for indoor localization and outperform a classical power level based ILS, LANDMARC, and a recently proposed ILS, SAIL. In addition, in the aspect of hardware cost, for a medium or large localization area, unlike SpotFi [3] that needed to deploy multiple access points (APs) with continuous power supplies, and LANDMARC, including its variants VIRE [12] and SAIL[11], which needed to deploy very expensive RFID readers to receive the signal of tags with relatively long distances, the proposed system just needs to deploy the corresponding number of cheap wireless nodes according to actual site area.
(2) This paper introduces the Markov theory to remove the fluctuation in power levels, which has not been addressed in the literature.
(3) We propose a priority-based pattern matching technique to find the most similar power level vector with fingerprint based localization methods. We found a noteworthy phenomenon that, even if two power level vectors are similar in Euclidean distance, their corresponding nodes may still be located at two far-away points in the constructed radio map. Thus, the conventional sequence matching algorithm, such as k-Nearest-Neighbor (kNN), is unsuitable to our case.

The rest of this paper is organized as follows. Section 2 presents the framework of TrackCC. This is followed by the background and problem in Section 3. Then, the implementation processes of the offline stage and online stage are presented in Section 4 and in Section 5, respectively. Section 6 illustrates the system implementation and shows experimental results. Section 7 concludes our work.

## 2. System Framework

**Figure 1:**
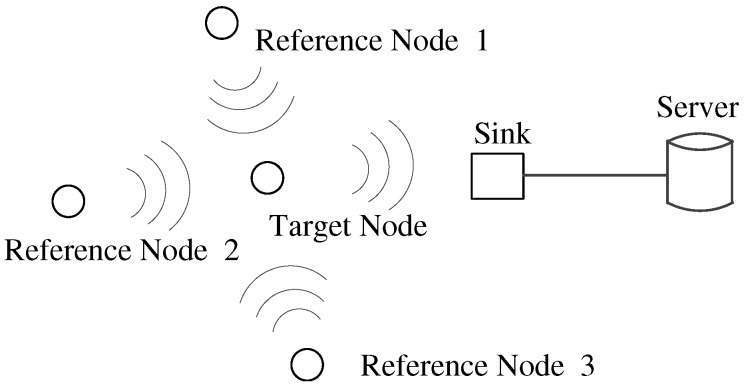
The network hardware infrastructure and logical communication links of TrackCC.

The proposed system consists of multiple wireless nodes and a server. According to requiring location information, nodes are classified into two kinds, target nodes and reference nodes. The network hardware infrastructure and logical communication links between these devices are illustrated in Figure 1.

In this figure, a target node needs to be localized and has the ability to receive reference nodes’ signals in real time and periodically forwards them to the sink node. All reference nodes have known locations. They do not communicate with each other, but broadcast data packets to the target. Because the target may receive data packets from multiple reference nodes, a reference node only sends essential data, such as its ID and current signal power level, to alleviate the traffic load at the target. The sink only receives data packets from the target, integrates data into a frame, and sends it to the server for further processing. The server is a workstation that is used as a center for data storing, frame processing and location estimation. Once a target is required to locate its position, the server will process its frame to calculate a position and give feedback on the localization result to specified users.

## 3. Background and Problem

The proposed system adopts reference nodes embedded with nRF24LE1 chips. It only supports four programmable signal propagation power levels (PL-1∼PL-4), where its maximum communication range under power level 0 dBm reaches 100 m if an external antenna is equipped [13]. It is noticed that the communication distance is comparable to that of a Wifi chip. We make a preliminary measurement about minimum power levels from reference nodes at multiple randomly selected testing points. According to measurement results, we observe that, for most of the testing points, the values of minimum power levels from the same reference node on the same position at different times may be rather different. This fact that received minimum power levels are variable over time is called “fluctuation”. Due to the impacts of electromagnetic interference (EMI) or internal multipath echoes [9], for a particular reference node, it is observed that the fluctuation phenomenas in received minimum power levels are very serious sometimes. It may derive more reliable and location-specific signal features from post-processing these measured data [14]. How to deal with such acute fluctuation has not been addressed in the literature.

Since reference nodes are required to periodically transmit four power level signals, the server only can provide one location estimation at every time slot. How to efficiently and correctly use a target vector of received minimum power levels and remove its fluctuation is a challenge for TrackCC to achieve high localization accuracy.

It is worth noticing that we found two candidates with the same Euclidean distance that may be located at two far-away positions on the constructed radio map. Thus, the conventional kNN-based sequence matching algorithm, which does not pre-build the classification model [15], becomes infeasible.

In summary, the acute fluctuation, the simple low-dimensional data and different positions with the same similarity challenge the fingerprint construction and affect the localization accuracy. The next few sections elaborate the solutions for the above problems.

## 4. Offline Stage

The offline stage is mainly used to form the radio map of the coverage area. We first place reference nodes at the grid points of the area. Then, based on collected vectors of received minimum power levels, fingerprints of the area are constructed. Here, a vector of received minimum power levels is abbreviated as VReMipl for the remainder of the paper.

### 4.1. Deployment

Without the loss of generality, we let *l* denote the number of supported power levels. Each reference node transmits signals powered with PL-1, PL-2, …, PL-*l*. We divide the transmission area of the node into two parts, a circle of radius $R_{1}$ and an annular area of outer radius $R_{2}$. For the former, because there is only one power level PL-1 available and its grain is coarse in terms of localization precision, this area is labeled as “invaluable location area” (ILA). Similarly, the latter is marked as a “valuable location area” (VLA). In VLAs, *l* signal power levels are distributed in the small distance from $R_{1}$ to $R_{2}$, which makes more precise distance estimation possible. We consider a deployment strategy that reference nodes are successively located at all grid points with grid side length $d^{\prime }=max\left\{min\left\{R_{2}-R_{1},2R_{1}\right\},R_{1}\right\}$. Under this deployment strategy, we have the following theorem.

**Theorem** **1.** *Suppose that reference nodes are placed at all grid points with grid area $d^{\prime }\times d^{\prime }$ in an approximately rectangle shaped target region with the length at least $3d^{\prime }$, where $d^{\prime }=max\left\{min\left\{R_{2}-R_{1},2R_{1}\right\},R_{1}\right\}$, and the ILA of any reference node can be covered by the VLAs of other reference nodes.*

**Proof:** See Appendix A.

Suppose that the transmission range increment between two adjacent power levels are $a_{1},a_{2},\cdots ,a_{l-1}$, consecutively. Then, we have $R_{2}-R_{1}=\sum_{i=1}^{l-1}a_{i}$. If the minimum value in set $\left\{a_{1},a_{2},\cdots ,a_{l-1}\right\}$ equals *d*, then *d* can be considered as the localization accuracy. It is because

(1) for any reference node, if the target moves more than *d* in its VLA, even in the case of open space, the value of received power level will be changed; and
(2) there may be d×d-sized square grids, where nodes’ movement will not result in changes in VReMipl value.

### 4.2. Remove Fluctuation

During the measurement process, we first locate one target node at each grid center point for a while to collect minimum power levels obtained from *n* different reference nodes. The detailed process is described as follows. Every reference node transmits signals periodically with a period of $h_{0}$, by powering in the following order: PL-1, PL-2,…, and PL-*l*. When the target is closer to a reference node, it may receive signals at multiple power levels. However, the target only records the minimum one, denoted by $PL_{min}$, and reports it to the sink. Since the communication chip used in this paper only supports several distinct programmable power levels, and it is not able to measure the RSSI value of the radio signal, traditional RSSI-based ILS solutions are not feasible in this case. Here, $PL_{min}$ is used to reflect the received signal strength at the grid point from the reference node. Supposing that $PL_{min}$ =PL-*i*, it is implied that the target node can receive the data packets sent by the reference node at the power from PL-*i* to PL-*l*. Therefore, the minimum power level actually represents all information involved in all power levels. Smaller *i* means that the received signal strength of the reference node at the location is stronger. For each time slot $h_{0}$, a data frame is formed in the format as below:

$$<t_{i},g_{j},\left(ref_{1},PL_{1,min}^{j,i}\right),\ldots ,\left(ref_{m},PL_{m,min}^{j,i}\right),\ldots ,\left(ref_{n},PL_{n,min}^{j,i}\right)>$$

where $t_{i}$ is the current time stamp (i∈Z), $g_{j}$ is the sequence number of grid center point, $ref_{m}$ is the reference node’s ID and $PL_{m,min}^{j,i}\left(m\in \left\{1,2,\cdots ,n\right\}\right)$ is the received minimum power level from reference node *m* at $t_{i}$. As mentioned earlier, even if in open space, not all frames formed during the period of collection may be identical. In order to facilitate the pattern matching in the online stage, removing fluctuation is required to obtain the fingerprint at grid point $g_{j}$.

For a given reference node $ref_{m}$, the target node at grid center point $g_{j}$ continuously records the minimum power levels from $ref_{m}$ along the time, denoted as $PL_{m,min}^{j,0}$, $PL_{m,min}^{j,1}$, ⋯, $PL_{m,min}^{j,i}$, ⋯. In order to represent the case that the target node does not receive $PL_{min}$ from a reference node at a certain time slot, a constant symbol Not-Receive (NR) is used. Such a recording process can be modeled as a discrete time discrete state stochastic process, denoted as $X_{m}^{j}\in S=\left\{s_{1},s_{2},\cdots ,s_{l},s_{l+1}\right\}$. Similar to [16], $X_{m}^{j}$ can be modeled as a Markov chain with a state space *S*, and the state transition matrix $P=\{P_{ij},i,j=1,2,\ldots ,l,l+1\}$, where $P_{ij}$ represents the transition probability from power level $s_{i}$ to $s_{j}$. $P_{ij}$ can be estimated by empirical measurements over a time period, which approximates the proportion of the number of transition $n_{ij}$ to the total number of all transitions starting from $s_{i}$, where $n_{ij}$ denotes transition from state $s_{i}$ at one time slot to state $s_{j}$ at the next time slot. $P_{ij}$ can be written as

(1) $$P_{ij}=\frac{n_{ij}}{\sum_{k=1}^{l+1}n_{ik}}.$$

From [17], since the samples of $X_{m}^{j}$ are along a discrete time, every state is accessible to other states, i.e., this chain is irreducible. In addition, this chain is not periodic due to the randomness of signal multipath fading and temporal dynamics. According to the theorem in [17], there always exists limiting probability in this chain. Define the state probabilities as $\Pi =\{\pi_{i},i=1,2,\ldots ,l,l+1\}$. Then, the state with the maximum probability is defined as the stable state of $X_{m}^{j}$, i.e., the stable power level for the reference node $ref_{m}$ at grid $g_{j}$. Π can be obtained by solving the following set of equations:

(2) $$\left\{\begin{array}{c} \pi_{j}=\sum_{i=1}^{l+1}\pi_{i}P_{ij}\;j=\left\{1,2,\cdots ,l,l+1\right\},\\ \sum_{i=1}^{l+1}\pi_{i}=1. \end{array}\right.$$

Once the stable states of *n* reference nodes are obtained at grid $g_{j}$, this grid’s fingerprint will be constructed by them in series. Finally, the fingerprints of all grids will be stored in the database stamped with the position of corresponding grid center points.

For *n* deployed reference nodes, when the server receives *f* frames for time $f\times h_{0}$ at grid $g_{j}$, it starts to construct the fingerprint for this grid. *f* frames are stored by row in a matrix $D_{j}$ based on FCFS (First Come, First Serve). In order to get $V_{m}=\{PL_{m,min}^{j,0},PL_{m,min}^{j,1},\cdots ,$$PL_{m,min}^{j,i},\cdots ,PL_{m,min}^{j,f}\}$, every $PL_{min}$ in $D_{j}$ will be added into a corresponding vector $V_{m}$ if its reference tag’s ID is *m*. Then, *n* Markov chains are modeled on the vectors $\left\{V_{m},m=1,2,\ldots ,n\right\}$. For a vector $V_{m}$, the calculation of matrix $P_{m}$ is as follows.

At the beginning, define three (l+1)-order matrices, called frequency count matrix *C*, state transition matrix *P* and summation vector *S*, which are initialized to zero. Then, vector $V_{m}$ is traversed. During the traversing process, if $V_{m}\left(i\right)$ is in state *a* and $V_{m}\left(i+1\right)$ is in state *b*, $C_{ab}$ will increase by one. Then, the total number of all transitions starting from $s_{i}$ is summed as $S_{i}$. Finally, based on (1), the value of $P_{ij}$ is the proportion of the value of $C_{ij}$ to the total frequency $S_{i}$. Once *P* is derived, set Π will be calculated based on (2). The state that has the maximum limiting probability is recorded as the stable state $f_{m}$ and $\left\{f_{m},m=1,2,\ldots ,n\right\}$ represents the fingerprint *F*. The detailed fingerprint construction algorithm is summarized in Algorithm 1.

In order to know how many frames are sufficient for evaluating *f*, we need to obtain the fluctuation condition in real time. Therefore, TrackCC first receives a few frames to avoid the estimation error caused by the accidental errors. Then, the above fluctuation removal procedure is executed once before receiving a new frame, until the maximum value of Π can be obtained.

As stated in Section 3, fluctuations exist in the most of the grids. If the fluctuation is not severe in a grid, the VReMipl may be the same for multiple frames. Thus, to further relieve the transmission overhead, we define the empirical threshold TH. Denote the number of same received frames to be $f^{\prime }$. If $\frac{f^{\prime }}{f}⩾TH$, no more frames are required and the same frame is considered as the fingerprint.

**Figure 2:**
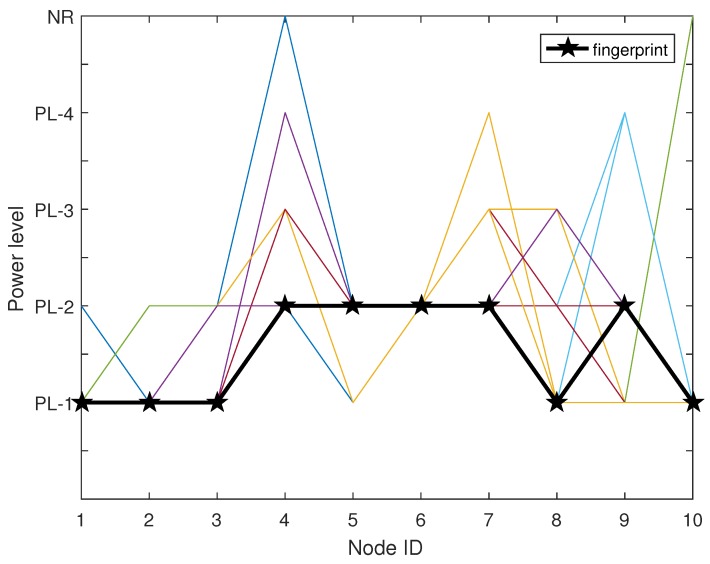
The resulting demonstration of removing fluctuation using Markov chain.

We now use an example as shown in Figure 2 to illustrate the fingerprint construction procedure. The figure is plot based on 66 pieces of measurement data in the 7th grid of our location area as shown in the experiment section, where there are 10 reference nodes deployed. The black line represents the fingerprint of this grid and colorful lines represent different frames in $D_{7}$. For example, for stable state of reference node 4 with four power levels, $f_{4}$, its 5 × 5-sized frequency count matrix *C*, state transition matrix *P* and state limiting probability set Π have been calculated, respectively, as

$$C=\left[\begin{array}{ccccc} 0 & 0 & 0 & 0 & 0\\ 0 & 56 & 3 & 1 & 1\\ 0 & 3 & 0 & 0 & 0\\ 0 & 1 & 0 & 0 & 0\\ 0 & 1 & 0 & 0 & 0 \end{array}\right],$$

$$P=\left[\begin{array}{ccccc} 0 & 0 & 0 & 0 & 0\\ 0 & 0.918 & 0.049 & 0.016 & 0.016\\ 0 & 1 & 0 & 0 & 0\\ 0 & 1 & 0 & 0 & 0\\ 0 & 1 & 0 & 0 & 0 \end{array}\right],$$

$$\Pi =\left[\begin{array}{ccccc} 0 & 0.924 & 0.045 & 0.015 & 0.015 \end{array}\right].$$

**Algorithm 1:** The construction of fingerprint for grid $g_{j}$
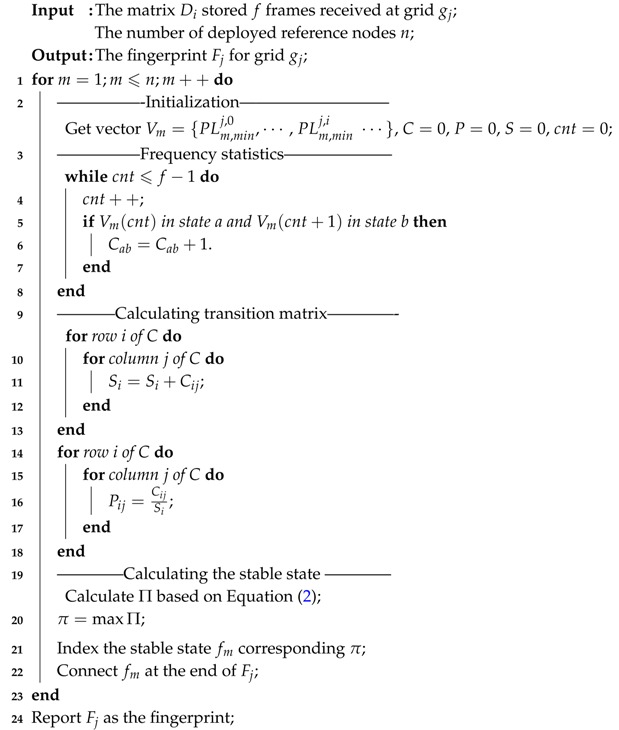



From matrix *C*, we can observe that there are 56 (the value of $C_{22}$) times that received minimum power levels from reference node 4 is PL-2. There are also three (the value of $C_{23}$) times fluctuating from PL-2 to PL-3. Clearly, fluctuation exists between PL-2, PL-3, PL-4, and even NR. However, because $\pi_{2}$ is the maximum one, the stable state is PL-2 after removing fluctuations. Finally, 10 stable states can be derived to represent the fingerprint.

## 5. Online Stage

The main task of the online stage is to provide position estimation for the target. The process is briefly described as follows. When the target node goes into the grid of a location area, it receives power levels from reference nodes and records $PL_{min}$ for each of them. Then, when the server receives a frame forwarded from the target, it extracts all power levels $PL_{min}$ and integrates them into a target vector by the ascending order of corresponding reference node’s ID $ref_{n}$. After that, a sequence classification scheme is applied to choose a fingerprint with the highest similarity. This procedure includes the similarity calculation between fingerprints and target vector and priority-based pattern matching. Finally, the position of that selected fingerprint is considered the target’s estimated position.

### 5.1. Similarity Calculation

Xing et al. [15] have pointed out that the distance function, which measured the similarity between sequences, determined the quality of distance-based sequence classification significantly. The target vector extracted from each frame is highly valuable in the similarity calculation process. More specifically, those elements PL-2–4 in the vector mean that the target tag is located in the VLA of the corresponding reference nodes. The drift (the effect of Dynamic Time Wrapping (DTW)) on the whole vector will make characteristics lost so that the accuracy of further pattern matching degrades seriously. Therefore, Euclidean distance, which is sensitive to small distortions on both axes, is chosen in this case. If the Euclidean distance is smaller, the similarity will be higher. Note that Keogh et al. [18] have shown that, when applying the 1NN classifier, Euclidean distance is surprisingly competitive in terms of accuracy compared to other more complex similarity measures.

Suppose that *n* reference nodes are deployed in the location area that is split into *G* grids. In the *i*th grid, the target vector $T_{i}$ and the fingerprint $F_{i}$ are

$$T_{i}=\left[\begin{array}{cccccc} T_{i}\left(1\right) & T_{i}\left(2\right) & \ldots & T_{i}\left(k\right) & \ldots & T_{i}\left(n\right) \end{array}\right],$$

$$F_{i}=\left[\begin{array}{cccccc} F_{i}\left(1\right) & F_{i}\left(2\right) & \ldots & F_{i}\left(k\right) & \ldots & F_{i}\left(n\right) \end{array}\right].$$

Then, their Euclidean distance is

(3) $$dist\left(T_{i},F_{i}\right)=\sqrt{\sum_{k=1}^{n}{\left(T_{i}\left(k\right)-F_{i}\left(k\right)\right)}^{2}}.$$

In TrackCC, we calculate the Euclidean distances between $T_{i}$ and all $\left\{F_{i},1⩽i⩽G\right\}$ and record the calculations in a candidate list CL by ascending order.

### 5.2. Priority-Base Pattern Matching

As stated above, the list CL contains position coordinates and their distances. If the Euclidean distance of the candidate with the highest similarity is zero, this candidate will be directly considered as the calculated position of the target. Otherwise, the priority-based 1NN technique will be adopted for the list CL to calculate elements’ priorities, and the candidate with the highest priority is then considered as the calculated position.

To facilitate the presentation, we only focus on multiple candidates with the same smallest Euclidean distance in list CL and ignore all others. Each candidate has an initial priority of 5. The lower the number, the higher the priority. The process of priority evaluation is described as follows:

(1) In accordance with their existing order in list CL, a candidate is selected as a benchmark, denoted with $CL_{B}$ .
(2) TrackCC records two power levels from the same reference node in the target vector and the benchmark, denoted as $PL_{T}$ and $PL_{B}$, respectively.
(3) The priority to $CL_{B}$ is changed based on a priority conversion rule from transition $PL_{T}$ to $PL_{B}$. The details of the priority conversion rule will be provided later. Then, return to step 2 until two vectors have been traversed.
(4) Return to step 1, pick another candidate as a new benchmark and go to step 2, until all candidates have been traversed.

During the above process, the priority conversion rule is a key. Before presenting its formulation, we first introduce the definition of Transformation as follows.

**Definition** **1.** *Transformation is defined as an action that the value of $PL_{T}$ needs to be changed to $PL_{B}$ in operation to remove fluctuations for the target vector T.*

When the target is located correctly, we investigate the transformation trend of power levels between a target vector and its true candidate. According to the records for specified transformation, the following hypotheses are verified correctly.

**Hypothesis** **1.** *If a power level transforms to itself, the priority will not change.*

**Hypothesis** **2.** *The probability that a power level transforms from PL-1 to others is lower than transforming reversely and the probability of transformations from NR to PL-1–l is the minimum.*

**Hypothesis** **3.** *The probability that a power level transforms to the next strong level is greater than transforming to the previous weak level in the range of PL-2–l.*

Based on the above hypotheses, we develop the priority conversion rules, as shown in the Table 1. From the table, based on Hypothesis 1, if PL-$1_{T}$–${\mathrm{NR}}_{T}$ transform to themselves, the priority to $CL_{B}$ will not change. Therefore, the numbers in the diagnoses are zeros. To implement Hypothesis 2, we set the rule in the PL-$1_{T}$ and ${\mathrm{NR}}_{T}$ rows. In the PL-$1_{T}$ row, after transformation from PL-$1_{T}$ to {PL-$i_{B},i=2,3,\ldots ,l\}$, the priority will be lower than that after transformation from {PL-$i_{T},i=2,3,\ldots ,l\}$ to PL-$1_{B}$. In the ${\mathrm{NR}}_{T}$ row, if NR transforms to {PL-$i_{B},i=1,2,\ldots ,l\}$, the priority will be reduced with the decrease of ${\mathrm{PL}}_{B}$. In addition, the priority after transformation from PL-$i_{T}$ to PL-${\left(i+1\right)}_{B}$ will be 1 higher than that after transformation from PL-$i_{T}$ to PL-${\left(i-1\right)}_{B}$, i=2,3,…,l. This takes Hypothesis 3 into account. The detailed priority-based 1NN pattern matching algorithm is given by Algorithm 2.


**Algorithm 2:** The priority-based 1NN pattern matching algorithm
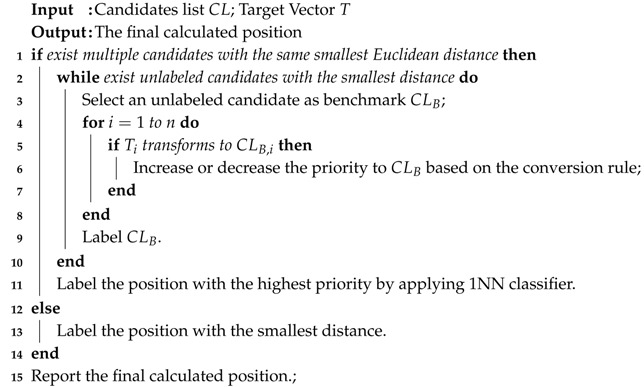



## 6. System Implementation and Experimental Results

**Figure 3:**
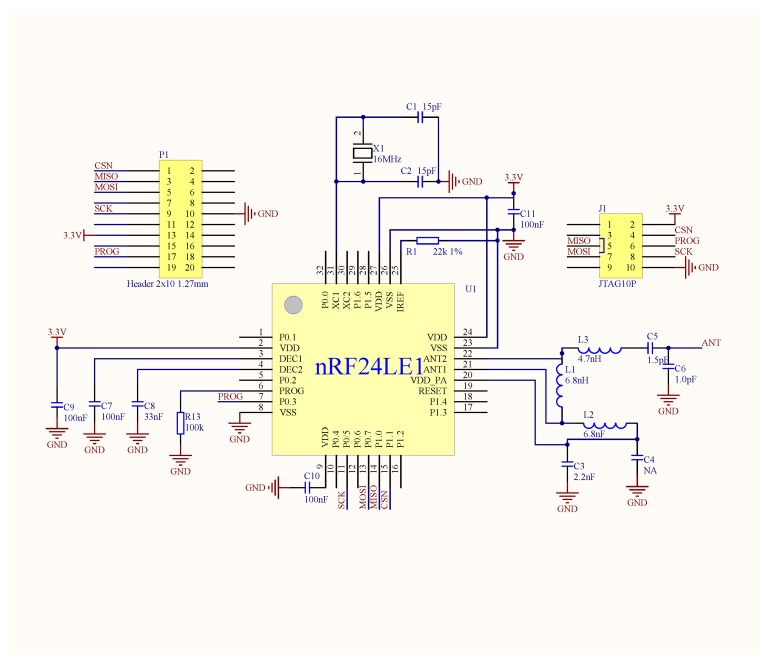
The key circuit diagram of the TrackCC hardware.

**Figure 4:**
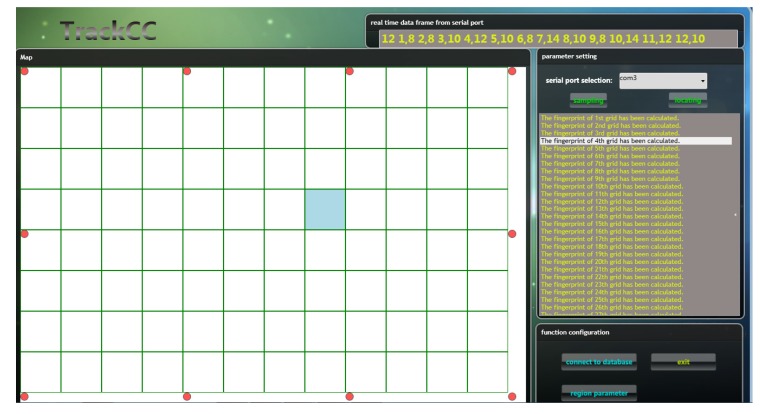
The software of the TrackCC running on the server.

We implemented a prototype system of TrackCC. In our implementation, every node integrates an nRF24LE01 SoC, which works normally on 3.3 V and equips a 4.2 V rechargeable Li-Battery. The corresponding key circuit diagram is shown in Figure 3. This diagram incorporates the usage of nRF24LE01’s pins and the design of critical peripheral circuits, such as FLASH programming interface and Peripheral board interface. Due to the gain effect of external antenna, the sink can receive a target’s signal within around 100 m. The server is a workstation equipped with Windows 7 operating system. A localization software encoded by C# runs on the server to implement the algorithm, as shown in Figure 4. For the convenience of coding and the chip’s configuration, we let the values of PL-1–4 correspond to 8, 10, 12, 14, respectively. We also let NR correspond to 16, instead of zero, to avoid sudden increment of Euclidean distance between two sequences.

**Figure 5:**
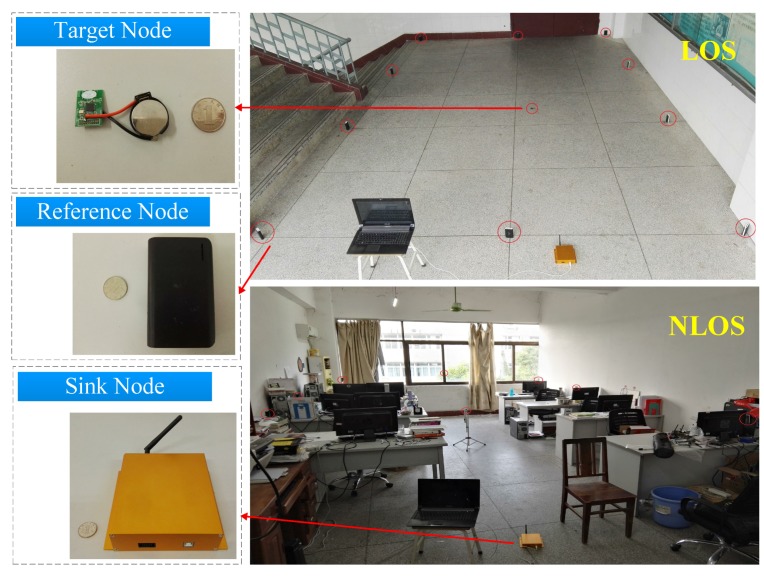
The experimental environment.

We deployed 10 reference nodes around a 6 m × 4 m space area for LOS and a 6 m × 4 m common office with some obstacles (include desks, chairs and computers) for NLOS, as shown in Figure 5. There are 2 m spacing between reference nodes. Under the condition that reference nodes and target nodes do not equip external antennas, the maximum transmission distance of PL-1 and PL-4 are measured as $R_{1}$ = 4 m and $R_{2}$ = 6 m, respectively, and the minimum localization accuracy is *d* = 0.5 m. Thus, the deployment area is divided into 96 grids in both LOS and NLOS.

According to the observation, receiving at least 10 frames in each grid can avoid estimation errors caused by accidental errors, and setting the threshold TH= 0.6 enables a few more grids in which almost no fluctuations exist to accelerate fingerprint construction. For other grids, up to 60 frames are adequate to obtain state transition probability matrices and a grid’s fingerprint. All frames received at two stages are recorded for further detailed performance evaluation. Every experiment is repeated 20 times to avoid statistical errors. The initial position of any target node is randomly selected and the target can move along any of eight directions for requiring consecutive location services. We define straight-line distance *e* between the true coordinate $\left(x_{t},y_{t}\right)$ and the calculated coordinate $\left(x_{c},y_{c}\right)$ as the position error as

(4) $$e=\sqrt{{\left(x_{c}-x_{t}\right)}^{2}+{\left(y_{c}-y_{t}\right)}^{2}}.$$

**Benchmarks:** Both LANDMARC [10] and SAIL [11] have been implemented as the comparison benchmarks, where the numbers of reference tags and readers used in LANDMARC and SAIL are the same as the number of grid center points and reference nodes in the TrackCC prototype system, respectively.

### 6.1. The Performance in the LOS Scenario

**Figure 6:**
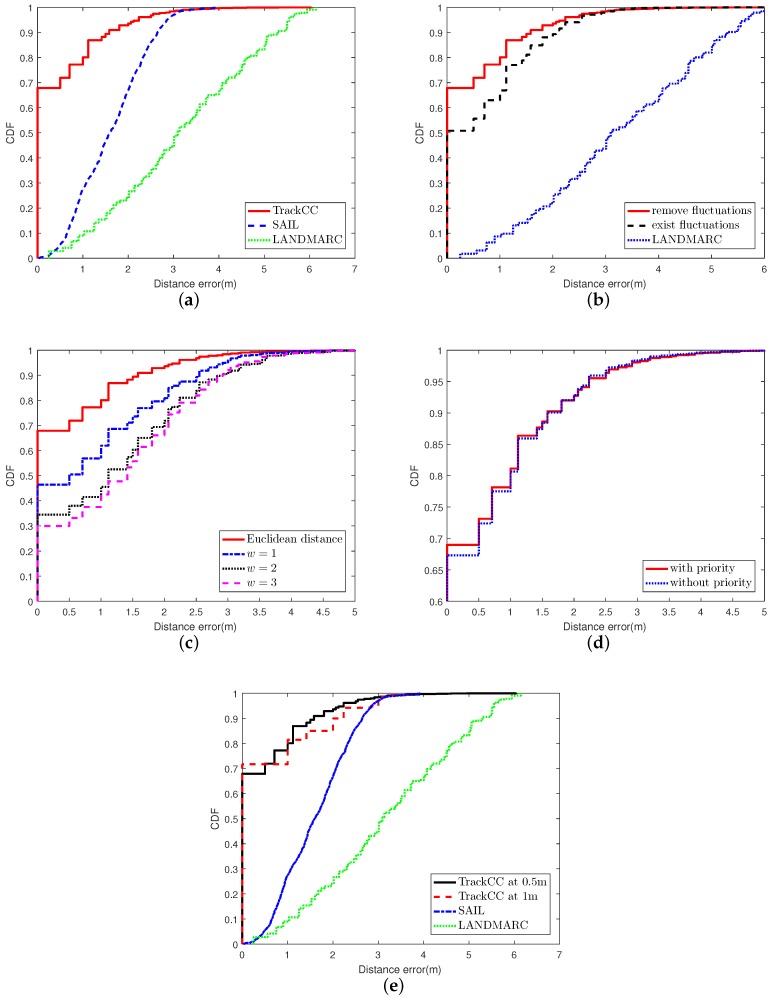
The performance evaluation of TrackCC in the case of line-of-sight (LOS). (**a**) the performance in the case of LOS; (**b**) fluctuation removal; (**c**) similarity calculation; (**d**) priority conversion rule; (**e**) expand grid to the size of 1 m × 1 m.

Figure 6a shows the cumulative distribution function (CDF) of position estimation errors for three ILSs, LANDMARC, SAIL and TrackCC. The performance is evaluated in the LOS scenario through three experiments. In the first experiment, we apply the fluctuation removal and the priority-based 1NN pattern matching techniques. From this figure, we can observe that the performance of TrackCC in terms of localization accuracy is much more superior, where 68% localization errors are lower than 0.5 m and 80% errors are lower than 1 m. In contrast, for SAIL, 5% of localization errors are lower than 0.5 m and the value for LANDMARC is less than 2.5%. The poor localization precision of LANDMARC and SAIL mainly owns to acute fluctuations of VReMipls. In addition, it is worth noticing that we found that two candidates with the same Euclidean distance may be located at two far-away positions in the constructed radio map. Thus, the conventional kNN based sequence matching algorithm, integrating LANDMARC and SAIL, is unsuitable for our situation.

### 6.2. The Effect of Fluctuation Removal

The fluctuation removal at the offline stage is a key step to constructing fingerprints with higher state possibility values. In the experiment in which the fluctuation removal is disabled, a grid’s fingerprint is constructed by randomly selecting a frame that was previously collected in that grid.

Figure 6b shows the cumulative distribution function (CDF) of position estimation errors with/without fluctuation removal. We can observe that the accuracy of correct localization with fluctuation removal is about 17% higher than that without fluctuation removal, and 63% higher than LANDMARC. For TrackCC without the fluctuation removal, we found that the probability of fingerprints in different grids being the same is 2–13% based on 20 groups of experiments with each containing 96 random samples. If the target vector is the same as multiple fingerprints, TrackCC will not provide a correct location estimation, and the accuracy will decrease almost 13% along with the increase in the number of location requisitions. On the contrary, through removing signal fluctuations, we observed that none of the fingerprints in the LOS or NLOS scenarios are identical. Namely, each fingerprint of the grid is unique. Therefore, even if the number of fingerprints becomes larger, their uniqueness is greatly enhanced after the fluctuation removal. In addition, these fingerprints can better reflect the real situation of signal strength in corresponding grids.

### 6.3. Euclidean Distance and DTW

As mentioned earlier, some distance function, such as DTW, is likely to distort, drift, stretch, or filter two series during the similarity calculation. This makes characteristics of sequences become lost. The goal of DTW is aligning two similar series and calculating the best distance by dynamic programming. It has a parameter window (w), which constrains the boundary of wrapping path. We implement the DTW algorithm for comparison and evaluate the effect of distortion as shown in Figure 6c. Note that Euclidean distance is replaced by DTW, while *w* is 1–4, respectively, in four experiments. We can observe that the precision using Euclidean distance is the best and much better than DTW. The effect of fluctuation removal disappears once the distortion is allowed. In addition, if w⩾3, the precision will change very little. It means that only a few characteristics, which are used by Euclidean distance to obtain the real similarity, have been wrapping.

### 6.4. The Performance of Priority Conversion Rule

The proposed priority conversion rule is an empirical one. The priority-based 1NN pattern matching technique can improve 2% in terms of accuracy, as shown in Figure 6d. Note that, since Hypothesis 3 is proposed based on the observation of correct location, this rule including Hypothesis 3 is beneficial to the improvement of the accuracy.

### 6.5. The Localization Effect for Different Sizes of Grids

In Section 4, it has been shown that the side length of grid d is the same as the localization accuracy. Thus, enlarging the grid will reduce the sampling time, but it will impact the resolution of the radio map, resulting in sparse radio map data. When the length of the grid is increased to 1 m, the CDF of position errors is shown in Figure 6e. We can observe that, when the grid becomes larger, the ability to correctly localize target nodes in their located grid cells can be improved. This is because decreasing the number of grids results in the reduction in the number of fingerprints, which directly leads to a drop in the probability of fingerprint matching errors. Therefore, in our experiment, 72% positioning errors are less than 1 m. However, once the positioning fails, the localization error will be greater than the length of grid (1 m), and the gradient of the increase in error becomes larger.

### 6.6. The Performance in the NLOS Scenario

**Figure 7:**
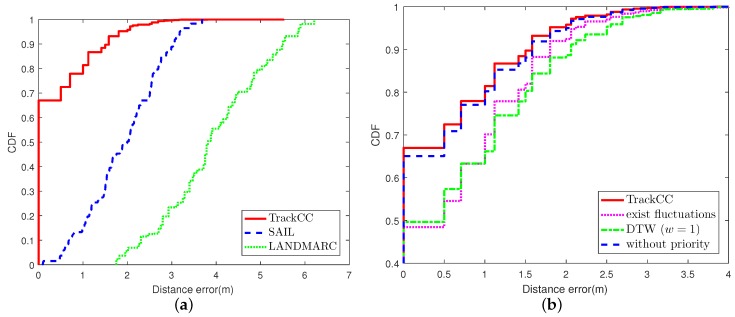
The performance evaluation of TrackCC in the case of non-line-of-sight (NLOS). (**a**) the performance in the case of NLOS ; (**b**) the detailed evaluation of NLOS scenario.

We also evaluate the performance of TrackCC in the NLOS scenario. The experiment process is the same as that in the LOS scenario. The CDF curves of overall performance and detailed evaluation are shown in Figure 7a,b, respectively. It is obvious that the accuracy is similar to that in the LOS scenario. This is beneficial from the whole area’s radio map that has taken the interference from obstacles into account at the offline stage. Imagining that if a target node receives PL-2 from a reference node on the side of a table, it may suddenly change to PL-4 due to a multipath effect after moving to the other side. Thus, LANDMARC may choose multiple far-away locations as the input of *k*NN, so that its accuracy is much lower than that in the case of LOS. While SAIL utilizes the fact that adjacent tags have high similarity in received signal strengths, the clustering result may not change greatly, although there may be changes in received minimum power level of tags. Hence, compared to the LOS scenario, its localization accuracy does not decline obviously in the case of NLOS.

## 7. Conclusions

This paper exploits a type of low-power and widely used commercial chip, nRF24LE1, for indoor localization, and proposes a novel estimation approach package, called TrackCC. Compared to RSSI based radio chips, the sensing node has obvious superiority in terms of cost, but has two inherent main drawbacks: (1) it has only four available power outputs so that directly applying existing distance estimation based strategies results in tremendously poor localization accuracy, and (2) it is extremely sensitive to environmental noise. To address these challenges, this paper adopts the fingerprint-based technique, which can perform well in both LOS and NLOS scenarios. In order to address the fluctuation issue, we exploit the Markov theory to construct a state transition matrix to remove the fluctuation. Then, by adopting the 1-nearest-neighbor (1NN) technique, we propose a priority-based pattern matching algorithm to search for the most similar match in the radio map to estimate the real position of unknown tags. Experimental results show that in both LOS and NLOS cases, TrackCC can guarantee that 68% localization errors are lower than 0.5 m. 

## Figures and Tables

**Figure A1:**
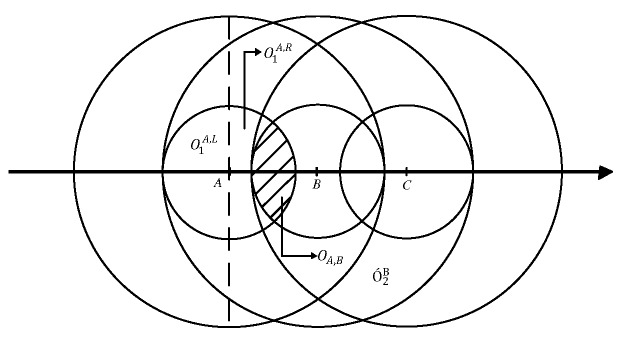
The illustration of proof of the second case

**Table 1:** The priority conversion rule.

		Benchmark Candidate						
		PL-1	PL-2	PL-3	PL-4	*…*	PL-l	NR
Target	PL-1	0	+2	+3	+4	*…*	+*l*	+(*l*+1)
	PL-2	-1	0	-2	+1	*…*	+(*l*-3)	+(*l*-2)
	PL-3	+1	-1	0	-2	*…*	+(*l*-4)	+(*l*-3)
	PL-4	+2	+1	-1	0	*…*	+(*l*-5)	+(*l*-4)
	⋮	⋮	⋮	⋮	⋮	⋱	⋮	⋮
	PL-*l*	+(*l*-2)	+(*l*-3)	+(*l*-4)	+(*l*-5)	*…*	0	−2
	NR	+(*l*-1)	+(*l*-2)	+(*l*-3)	+(*l*-4)	*…*	−1	0

## Appendix A

**Proof of Theorem 1:**

For the purpose of explanation, we consider a diagram as shown in Figure A1. We let $O_{1}^{A}$ denote reference tag *A*’s ILA, $O_{1}^{A,L}$ and $O_{1}^{A,R}$ denote the left and the right half-circle of $O_{1}^{A}$, respectively. Let ${\overset{´}{O}}_{2}^{B}$ denote the VLA of reference tag *B*, and $O_{2}^{B}$ denote its ILA. Similarly, define ${\overset{´}{O}}_{2}^{B,L}\left(O_{2}^{B,L}\right)$ and ${\overset{´}{O}}_{2}^{B,R}\left(O_{2}^{B,R}\right)$ as the left and the right half circle of ${\overset{´}{O}}_{2}^{B}\left(O_{2}^{B}\right)$, respectively. Two different cases are discussed as follows.

(1) When $2R_{1}⩽R_{2}-R_{1}$, we have $R_{2}⩾3R_{1}$. Since $d^{\prime }=max\left\{min\left\{R_{2}-R_{1},2R_{1}\right\},R_{1}\right\}$, we have $d^{\prime }=2R_{1}$, and the radius of $O_{1}^{A}$ and $O_{1}^{B}$ are $R_{1}$ . It is easy to get that $O_{1}^{A}$ and $O_{1}^{B}$ are two circumscribed circles. Define that the overlapping area of $O_{1}^{A}$ and $O_{1}^{B}$, denoted with $O_{A,B}$, equals ϕ. For $O_{2}^{B}$ of radius $R_{2}$ centered at *B*, even in the case $R_{2}=3R_{1}$, it also contains $O_{1}^{A}$ and $O_{1}^{B}$. Since ${\overset{´}{O}}_{2}^{B}=O_{2}^{B}-O_{1}^{B}$ and $O_{1}^{A}\cap O_{1}^{B}=O_{A,B}=\phi$, we can derive $O_{1}^{A}\subseteq {\overset{´}{O}}_{2}^{B}$. Similar with the above derivation, reference tag B’s ILA can also be covered by A’s VLA. In the case of $2R_{1}⩽R_{2}-R_{1}$, the theorem holds as long as there exist at least two tags in a row in the target region.
(2) When $2R_{1}>R_{2}-R_{1}$ , from $d^{\prime }=max\left\{min\left\{R_{2}-R_{1},2R_{1}\right\},R_{1}\right\}$, it is easy to derive that $d^{\prime }=R_{2}-R_{1}<2R_{1}$. Since the radius of $O_{1}^{A}$ and $O_{1}^{B}$ are $R_{1}$, and $d^{\prime }<2R_{1}$, $O_{1}^{A}$ and $O_{1}^{B}$ overlap, i.e., $O_{A,B}\neq \phi$. For the two circles $O_{1}^{A}$ and $O_{2}^{B}$ with $d^{\prime }=R_{2}-R_{1}$ meters away from their centers, because the radius of $O_{2}^{B}$ and $O_{1}^{A}$ are $R_{2}$ and $R_{1}$, respectively, we can obtain that $O_{1}^{A}$ is contained in $O_{2}^{B}$, i.e., $O_{1}^{A}\subset O_{2}^{B}$. Furthermore, $O_{1}^{A}-O_{1}^{B}\subset O_{2}^{B}-O_{1}^{B}$. It is equivalent to ($O_{1}^{A}-O_{1}^{A}\cap O_{1}^{B})\subset \left(O_{2}^{B}-O_{1}^{B}\right)$. Thus, $O_{1}^{A}-O_{A,B}\subset {\overset{´}{O}}_{2}^{B}$. Similar to the above derivation, we have $O_{1}^{B}-O_{B,C}\subset {\overset{´}{O}}_{2}^{C}$. Since the minimum value of $d^{\prime }$ is $R_{1}$, according to the geometry theory, when $d^{\prime }$ belongs to the interval $\left[R_{1},2R_{1}\right)$, it is not hard to derive that $O_{A,B}\subset O_{1}^{B,L}$, and $O_{B,C}\subset O_{1}^{B,R}$. It means that $O_{A,B}\cap O_{B,C}=\phi$. Thus, $O_{A,B}\subset \left(O_{1}^{B}-O_{B,C}\right)\Rightarrow O_{1}^{A}-\left(O_{1}^{B}-O_{B,C}\right)\subset O_{1}^{A}-O_{A,B}\Rightarrow O_{1}^{A}=\left\{\left(O_{1}^{A}-\left(O_{1}^{B}-O_{B,C}\right)\right)\cup \left(O_{1}^{B}-O_{B,C}\right)\right\}\subset \left\{\left(O_{1}^{A}-O_{A,B}\right)\cup \left(O_{1}^{B}-O_{B,C}\right)\right\}\subset ({\overset{´}{O}}_{2}^{B}\cup {\overset{´}{O}}_{2}^{C}).$ Finally, we get that *A*’s ILA is covered by the union set of the VLAs of both tags *B* and *C*, which are two consecutive tags located on the right side of *A* as shown in Figure A1. Likewise, the claim holds true for the case on the left side. In the case of $2R_{1}>R_{2}-R_{1}$, it can be summarized that the theorem holds as long as there exist at least two continuous adjacent tags either on the left side or the right side.

Because the length of the rectangular target region is no less than $3d^{\prime }$, there are four deployed tags in a row. Therefore, for any tag, there must exist at least two tags deployed on the left or right side of it. Therefore, the theorem holds in both cases.   ☐
